# Innovative Gerontology in Higher Education: Transformative Effects of the Pandemic

**DOI:** 10.1093/geroni/igab046.2215

**Published:** 2021-12-17

**Authors:** Pamela Saunders, Yoon Chung Kim, Debra Dobbs

## Abstract

Gerontology in higher education is experiencing an exciting inflection point rising from the COVID pandemic pushing us to adapt our teaching modalities. Many educators have developed innovative learning experiences making use of creativity, virtual reality, online discussion boards, virtual tours, Jam Boards, videos, and breakout rooms. This symposium will bring together gerontologists and educators to discuss their educational innovations. Dr. Saunders will discuss the use of virtual reality in a Geriatrics clerkship experience to enhance knowledge, empathy, and attitudes towards older adults. Dr. Hanna and Ms. Kim will present the use of a virtual avatar to explore aging identity. Professor Barsness will discuss the participation of older adults from the community as subject matter experts. Ms. Redlich will share her virtual internship experience of exploring the intellectual and social benefits of adult study abroad. Although the pandemic was challenging to gerontological education, substantial transformations have been accomplished. The innovations described in this session broadened engagement of students with older adults to identify their strengths and challenges to flourish in the "New Normal."

